# 

**Published:** 1910-05

**Authors:** 


**Sixty-fifth Year.**

Vol. LXV. MAY, 1910. No. 10

### BUFFALO MEDICAL JOURNAL

**Established 1845 by AUSTIN FLINT, M. [illegible]**

LIBRARY
SURGEON GENERAL'S OFFICE
APR. 30 1910

**EDITOR:**

WILLIAM WARREN POTTER, M. D.

**ASSISTANT EDITORS:**

NELSON W. WILSON, M. D. JAMES WRIGHT PUTNAM, M. D.

**ASSOCIATE EDITORS:**

ERNEST WENDE, M. D. JOHN A. RAFTER, M. D.
JOHN PARMENTER, M. D. JOHN A. MILLER, Ph. D.
MAUD J. FRYE, M. D.

